# Nutritional modulation of the gut microbiome in allogeneic hematopoietic stem cell transplantation recipients

**DOI:** 10.3389/fnut.2022.993668

**Published:** 2022-10-20

**Authors:** Edoardo Muratore, Davide Leardini, Francesco Baccelli, Francesco Venturelli, Arcangelo Prete, Riccardo Masetti

**Affiliations:** ^1^Pediatric Oncology and Hematology “Lalla Seràgnoli,” IRCCS Azienda Ospedaliero-Universitaria di Bologna, Bologna, Italy; ^2^Department of Experimental, Diagnostic and Specialty Medicine (DIMES), University of Bologna, Bologna, Italy; ^3^Department of Medical and Surgical Sciences (DIMEC), University of Bologna, Bologna, Italy

**Keywords:** gut microbiota, hematopoietic stem cell transplantation, nutrition, diet, enteral nutrition, prebiotics, lactose

## Abstract

Allogeneic hematopoietic stem cell transplantation (allo-HSCT) represents a potentially curative strategy for many oncological and non-oncological diseases, but it is associated with marked morbidity and mortality. The disruption of gut microbiota (GM) eubiosis has been linked to major allo-HSCT complications, including infections and acute graft vs. host disease (aGvHD), and correlates with mortality. This increasing knowledge on the role of the GM in the allo-HSCT procedure has led to fascinating ideas for modulating the intestinal ecosystem in order to improve clinical outcomes. Nutritional strategies, either by changing the route of nutritional supplementation or by administering specific molecules, are increasingly being considered as cost- and risk-effective methods of modulating the GM. Nutritional support has also emerged in the past several years as a key feature in supportive care for allo-HSCT recipients, and deterioration of nutritional status is associated with decreased overall survival and higher complication rates during treatment. Herein we provide a complete overview focused on nutritional modulation of the GM in allo-HSCT recipients. We address how pre transplant diet could affect GM composition and its ability to withstand the upsetting events occurring during transplantation. We also provide a complete overview on the influence of the route of nutritional administration on the intestinal ecosystem, with a particular focus on the comparison between enteral and parenteral nutrition (PN). Moreover, as mounting evidence are showing how specific components of post-transplant diet, such as lactose, could drastically shape the GM, we will also summarize the role of prebiotic supplementation in the modulation of the intestinal flora and in allo-HSCT outcomes.

## Introduction

Allogeneic hematopoietic stem cell transplantation (allo-HSCT) represents a potentially curative strategy for many oncological, hematological, metabolic and immunological diseases (1–4). Complications, such as graft-vs.-host disease (GvHD) and infections, remain a major cause of morbidity and mortality, limiting the broader applicability of the procedure (5, 6). Patients undergoing allo-HSCT are subjected to high dose of chemotherapy, radiation, and antibiotics within a short time frame, that lead to mucosal barrier disruption and microbiota dysbiosis, characterized by reduced diversity, loss of commensals and expansions of potentially pathogenic bacteria (7–10). The degree of GM injury, and the establishment of specific GM signature are associated with major adverse outcomes. Reduced alpha diversity at engraftment is independently associated with increased mortality after transplantation (11). Overgrowth of *Enterococcus* is a risk factor for the development of aGvHD and for increased GvHD-related mortality and all-cause mortality (12), while *Blautia* is considered a protective factor from lethal GvHD (13). Intestinal domination, that occurs when a single bacterial taxon comprises 30% or more of the entire GM, is associated with the occurrence of blood stream infections (BSI) (14). Immune reconstitution, hepatic sinusoidal obstruction syndrome, febrile neutropenia, pulmonary complications and relapse of the primary disease have also been associated with intestinal microbiota composition after and/or before transplantation in single or multicenter studies (15–19). The biology underpinning the complex interplay between the GM, immune system and intestinal microenvironment has been partially addressed (15, 20–23) and needs to be fully elucidated (24). Gut microbiome-derived metabolites, among other factors, play a key role in mediating relevant biological processes during allo-HSCT. Such metabolites, particularly short chain fatty acids (SCFA), aryl hydrocarbon receptor ligands and bile acids, can protect intestinal epithelial cell from the damage occurring during transplantation and modulate the mucosal immunity reducing alloreactivity (23, 25). The increasing knowledge on the role of the GM during allo-HSCT has led to the fascinating idea of modulating the intestinal ecosystem toward a eubiotic configuration to improve clinical outcomes. Various interventions, both nutritional and non-nutritional, could be implemented with the aim of preserving GM homeostasis from the injury occurring during HSCT and/or restoring the GM diversity and composition after allo-HSCT (26). Among non-nutritional interventions, an antibiotic-sparing approach can help to preserve eubiosis (27, 28). “Direct” modulatory interventions using live microorganisms could also be applied, ranging from probiotic administration to fecal microbiota transplantation (FMT) (10). FMT directly modifies GM composition and has been used in HSCT setting, for the treatment of recurrent *Clostridioides difficile* infections and steroid-resistant gut GvHD or as preventive strategy in order to prevent dysbiosis (29).

Diet has also emerged in the past several years as a key candidate for the modulation of the GM, with the aim of affecting microbial composition, metabolic profile and ability to withstand the upsetting events occurring during transplantation. A profound link exists between nutrients and the GM. Diet is considered a major driver of the species and functions that reside within the GM (30). Even short-term changes in diet could rapidly affect the GM (31). For example, high-fiber and high-fermented-food consumption improves GM diversity, reduces inflammatory markers and has a distinct impact on immune response (32). Dietary interventions are now considered a key component in the management of several GM-related disease, with the main example being inflammatory bowel disease (33). Moreover, nutritional support is increasingly considered a key feature in supportive care for allo-HSCT recipients, as deterioration of nutritional status is associated with decreased overall survival and higher complication rates during treatment (34). In this review, we aim to summarize the present evidence regarding nutritional modulation of the GM in allo-HSCT recipients, ranging from changing the route of nutritional supplementation to administering specific molecules. We will also provide an in depth-focus on the potential mechanism involved in the complex relationship between diet, GM and clinical outcomes, underlining the most promising fields of research in the near future.

## The relationship between nutritional status and gut microbiota

Nutritional status can influence allo-HSCT outcomes. Serum albumin deficiency prior to transplant is associated with increased non-relapse mortality (35), and malnutrition, accounting both low BMI and weight loss, is considered a risk factor for severe aGvHD (36). On the other hand, conflicting data exist regarding the relationship between obesity and clinical outcome, but the majority of studies points toward a net negative effect (37).

Nutritional status and GM have a bidirectional relationship. Disturbances in microbiome affect the risk for under nutrition and obesity through the alteration of bacterial metabolites production, and malnutrition alters GM function and composition (38–40). In particular, the reduction in microbial gene richness increases along with the severity of obesity and its metabolic complications, including type 2 diabetes mellitus (41–43). Specific taxonomic and functional markers are associated with increased BMI and glucose metabolism deterioration, such as enrichment in Bacteroides enterotype 2 and impairment of biotin metabolism (38, 44), but further studies are needed to better decipher the complex relationship between obesity and the GM.

In the HSCT setting, the impact of obesity on the GM and GvHD pathogenesis was assessed both in mouse models and humans. In the mouse model, mice with diet-induced obesity had increased incidence of severe, rapid onset gut aGvHD with high lethality. Gut aGvHD in obese mice was mediated by donor CD4 + T cells and occurred even with a minor MHC incompatibility. Obese mice presented also increased epithelial cell apoptosis, gut permeability, endotoxin translocation across the gut, and radiation- induced gastrointestinal damage after conditioning. Moreover, increased proinflammatory cytokine production and expression of the GvHD marker ST2 (IL-33R) and MHC class II molecules was observed. In the same study, the human obese cohort (BMI > 30) showed higher transplant-related mortality at 1-year, worse histological gut GvHD severity and higher serum ST2 concentrations compared with non-obese transplant recipients. GM analysis pre-transplant, both in human and mice, revealed reduced GM diversity and decreased Clostridiaceae abundance in obese patients, particularly with lower abundance of genus *Clostridium*. In the mouse model only, the relative abundance of *Enterococcus* and *Akkermansia muciniphila* significantly increased in obese after transplantation. Therefore, obesity-associated GM alterations may render the patient more prone to gut epithelial damage, inflammation and gut aGvHD. Interestingly, prophylactic antibiotic treatment in obese mice improved gut GvHD histological severity and mortality, as well as reduced endotoxin translocation across the intestinal epithelium and inflammatory cytokine production, but did not protect against the development of cGvHD of the skin, highlighting the possibility of modulating obesity-associated dysbiosis (37).

Indeed, body mass composition itself could impact HSCT outcome, and GM composition could also influence the complex metabolic pathways regulating body composition. Muscle mass is an independent predictor of survival after HSCT, with sarcopenia associated with worse disease-free and overall survival (45, 46). The GM, and particularly its metabolites, play an important role in muscle metabolism, affecting skeletal muscle mass and function (47). Administration of soy-whey blended protein for 2 months in HSCT recipients who failed to improve muscle function within half a year resulted in significantly improved muscle area and muscle strength. However, in a small number of patients, muscle status did not improve. GM Alpha diversity significantly increased in responder to treatment, whereas it did not in non-responders. Moreover, abundance of most of the butyrate- producing taxa decreased significantly in non-responders. This important SCFA is known to regulate skeletal muscle energy expenditure and metabolism (48). *Ruminococcus* and *Veillonella* abundance correlated positively with muscle status, whereas *Streptococcus* correlated negatively (49). *Ruminococcus* species can metabolize monosaccharides and degrade mucin, producing acetic acid and formic acid (50). *Veillonella* enhance muscle function by converting lactic acid produced by muscles to propionic acid (51). Amino acid biosynthesis and pentose phosphate pathways were also higher in the GM of responders. Bacteria can enhance protein anabolism in the muscle by increasing amino acid bioavailability (52). Moreover, the pentose phosphate pathway is important for anabolic processes in the initial phases of skeletal muscle regeneration (53). These finding underline the important role of the GM on muscle metabolism after HSCT.

## The impact of nutritional route of administration on gut microbiota

Oral intake in the early post-transplantation period is severely impaired due to the side effects of the conditioning regimen, mainly vomiting, and mucositis. Parenteral nutrition (PN) has been historically used as supportive measure in order to avoid the deterioration of nutritional status in HSCT recipients (54–56). However, PN is associated with several metabolic, hepatic and intestinal complications. In recent years, enteral nutrition (EN) has been increasingly used in the HSCT setting considering the feasibility and clinical benefits of this approach (57, 58). EN is currently recommended by international guidelines as first-line nutritional support in transplant recipients if oral intake is insufficient (59). Nevertheless, PN is still largely adopted in the majority of transplantation centers, mainly due to patients and staff preference for PN and concerns regarding EN tolerance (60, 61).

The clinical positive effects of EN for nutritional support during the neutropenic phase after HSCT have been showed in several studies. In our recent meta-analysis the use of EN was associated with lower rates of aGVHD, aGVHD grade III-IV and gastrointestinal aGVHD compared to PN (62). Some studies also observed reduced infective complications, such as BSI (63). Interestingly, a survey in the Australian HSCT centers identified as the major barrier to the use of EN the perception of invasiveness and the perception of poor tolerance (58). These limitations should be directly addressed in future studies to clearly define the clinical benefit of EN.

**FIGURE 1:**
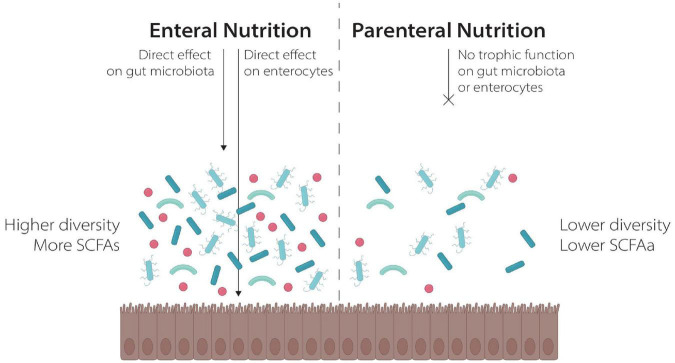
The effect of Enteral and Parenteral Nutrition on the gut ecosystem.

One possible explanation for these findings is the different effects of the two nutritional strategies on GM composition, as already demonstrated in different clinical and preclinical settings. On one side, PN reduced intestinal transit with subsequent GM dysbiosis and mucosal atrophy. It also determines a proinflammatory state with resulting loss of epithelial barrier function that leads to microbial translocation and bacteremia. On the other side, EN allows the maintenance of gut transit and seems to improve mucosa integrity. EN exerts a trophic effect on enterocytes, either directly providing nutrients in the gut lumen, or indirectly *via* the production of SCFA from the GM (64, 65). Moreover, EN seems to be essential to maintain an healthy gut mucosal and high GM diversity, as showed in preclinical and clinical models (64–68) (Figure 1). In murine models, PN results in reduced bacterial diversity (68) and a shift of microbiome to *Bacterioidetes* after several days of exclusive PN administration was noted (69, 70). Furthermore, a loss of bacterial diversity was observed in a neonatal pig model feed with PN (71).

Data on humans are limited, but seems to demonstrate that PN reduces GM diversity and is associated with an increased relative abundance of *Proteobacteria* (particularly *Enterococcaceae*), that proliferate in a nutrient-deprived environment, whereas a reduction in *Firmicutes* is observed, that seem to dominate when a valid nutrient supply is guaranteed (68, 72). The effect of PN on the GM of HSCT recipients was investigated in two large observational studies. The first one, on 80 HSCT recipients, groups with different GM diversity showed no differences in term of PN administration, with similar percentages of patients receiving PN during the pre-engraftment period in the two groups (73). Differently, in a second study investigating the relationship between GM and GvHD on 115 patients, patients receiving PN for a shorter period (<10 days) showed a higher abundance of genus *Blautia* compared to those receiving PN for a longer duration. Notably the inverse association of PN and *Blautia* was maintained even for patients who did not receive anaerobe-active antibiotics (13) *Blautia* is an anaerobic commensal producer of SCFA that has been associated with reduced GvHD related mortality (13).

To date, two studies specifically investigated the impact of EN on GM composition compared to PN in HSCT setting. In a cohort of 23 adult HSCT recipients randomly allocated to receive EN or PN, a shotgun metagenomic sequencing analysis of stool samples 30 days post-transplant, revealed no difference in term of microbial diversity in the two groups. However, significant differences in GM composition were reported in patients receiving predominantly EN compared to patients receiving predominantly PN. Particularly, the former presented a higher abundance of taxa associated with increased SCFA production, including *R. bromii*, *R. inulinivorans*, *A. hadrus*, and several *F. praunitzii* species. In the PN group, a greater abundance of *Enterococcus* and *Proteobacteria*, such as *Klebsiella*, was noted. Furthermore, patients who maintained higher levels of oral intake during the phase of nutritional support, whether parenteral, enteral or a combination of both, present significantly different GM composition with higher microbial diversity and relative abundance of SCFA-producing taxa, including *F. prausnitzii_C*, *R. inulinivorans*, and *Blautia.* A greater abundance of potential pathogens, such as *Klebsiella_A*, *Staphylococcus*, and *Enterococcus* was observed instead in patients who maintained a longer duration of minimal oral intake. Unfortunately, the small sample size and the absence of pre-transplant sampling limit the reliability of these findings (74). We reported a positive effect of EN on GM composition after HSCT in pediatric HSCT recipients. We assessed GM composition at the baseline, close after transplantation and during the immunological recovery following the HSCT. Patients receiving EN showed lower GM injury and an almost complete recovery of the diversity after HSCT. On the other side, patients receiving PN presented a microbial shift toward a dysbiotic profile and never achieved a return to pre-transplant microbial status. Some genera, including *Faecalibacterum, Dorea*, *Blautia, Bacteroides*, *Parabacteroides*, and *Oscillospira*, were relatively more abundant in EN patients after HSCT, confirming the higher presence of SCFA-producing bacteria in EN-treated patients. As expected, the fecal levels of SCFAs were restored to baseline values only in the EN group (75). In a recent study in adult HSCT recipients receiving EN, an analysis of intestinal microbiome was performed in a small subgroup of patients who received EN before conditioning. Interestingly, in two patients who discontinued EN and oral ingestion and shifted to PN, a drastic increase of *Enterococcaceae* was observed (76).

## The impact of specific dietary elements on gut microbiota

**FIGURE 2:**
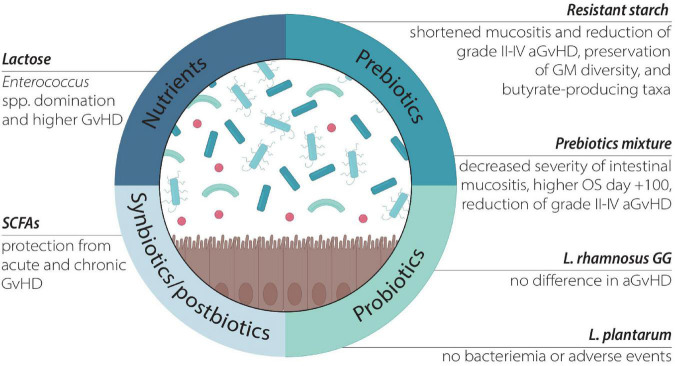
The effect of different nutritional compounds targeting the GM.

As mentioned, diet represents one of the most important ways to modulate the GM and has been increasingly studied (41, 77). On the other side, less is known on the effect of single dietary elements on GM. Some recent reports evaluated the impact of several dietary elements and nutritional strategies on allo-HSCT outcomes. These includes the role of nutrients, prebiotics, as well as the oral administration of probiotics, synbiotics, postbiotics, and lactoferrin (Table 1 and Figure 2).

**TABLE 1:** Summary of the main studies on GM modulation in allo-HSCT.

References	Dietary compound	Setting	Effect on GM	Effect on outcomes
Stein-Thoeringer et al. (12)	Lactose	Adult allo-HSCT	*Enterococcus* spp. domination.	Reduction of overall survival and increase of aGvHD
Iyama et al. (82)	Glutamine, fiber and oligosaccharides	Adult allo-HSCT	Reduction of translocating *Enterococcus* species	Decreased severity of intestinal mucositis (↓ days of diarrhea grade 2-3-4, *p* < 0.001, ↓ days of mucositis grade 3–4, *p* = 0.033) and increased overall survival at day + 100 (100 vs. 77%, *p* < 0.001).
Yoshifuji et al. (83)	Resistant starch and prebiotics containing glutamine, polydextrose, and lactosucrose	Adult allo-HSCT	No difference in GM composition and diversity.	Shortened duration of oral mucositis (median 11 vs. 14 days (*p* < 0.001) and diarrhea (7 vs. 9 days, *p* < 0.049). Reduction of cumulative incidence of grade II-IV aGvHD (14.3 vs. 43.8% *P* = 0.093)
Rota et al. (88)	Lactoferrin	Adult allo-HSCT	Not reported	Resolution of gut GvHD.
Gorshein et al. (100)	*L. rhamnosus GG (10 billion/day)*	Adult allo-HSCT	GM composition wasn’t affected by supplementation.	No difference in the incidence of GvHD (evaluated at 3-months intervals, p = 0.38).
Ladas et al. (96)	*Lactobacillus plantarum*	Children and adolescent allo-HSCT	GM colonization with administration of *Lactobacillus plantarum (orally or enteral feeding tube at 1* × *108 colony-forming units/kg/day, from day -8 to* + *14)*	No cases of *Lactobacillus plantarum* bacteremia (*n* = 0/30 patients).

### Nutrients and prebiotics

**FIGURE 3:**
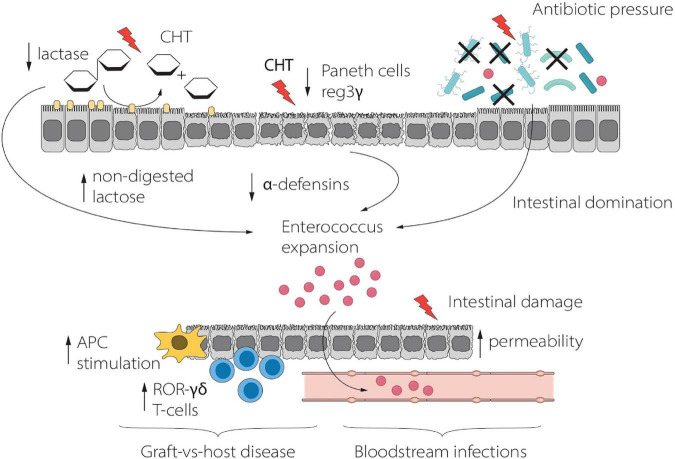
Schematic representation of the relationship between Lactose, *Enterococcus* and clinical outcome during HSCT. *Enterococcus* expansion, driven by lactose, promotes inflammation and GvHD, whereas translocation through the damaged intestinal mucosa results in BSI. APC, Antigen Presenting Cell; CHT, Chemotherapy.

Stein-Thoeringer et al. evaluated the role of lactose in the setting of allo-HSCT (12). 16S ribosomal RNA gene sequencing was used to study the fecal microbiota of 1,325 adult allo-HSCT recipients. Fecal *Enterococcus* spp. domination was found in up to 65% of patients after allo-HSCT and was associated with a significant reduction of overall survival and an increased risk of moderate-to-severe acute GVHD. Given that the growth of enterococci depends on lactose availability *in vitro*, the authors found that luminal lactose modulates the post-transplant *Enterococcus* overgrowth and GM recovery in both mouse models and *in vivo* (12). In particular, dietary lactose depletion in mice mitigated enterococcal expansion and reduced the severity of GvHD, with consequent improved survival. In humans, administration of broad-spectrum antibiotics generated *Enterococcus* overgrowth both in lactose absorbers and in patients carrying lactose-non-absorber genotypes, but *Enterococcus* domination was significantly prolonged in malabsorbers after antibiotics exposure (12). It is possible to speculate that enterocytes damage due to chemo-radiotherapy toxicity and alloreactivity induces loss of lactase and consequent increased luminal concentrations of lactose, which in turn is critical as a growth substrate for *Enterococcus* expansion. These findings might suggest that non-antibiotic-based interventions, such as lactose-free diet, could be a useful strategy to mitigate fecal pathobiont domination, possibly improving clinical outcome by reducing aGvHD and BSI in allo-HSCT recipients (Figure 3).

Prebiotics are defined as “a substrate which is selectively utilized by host microorganism conferring a health benefit” (78). They are selectively fermentable, non-digestible carbohydrates such as resistant starch, and in particular potato starch, fructo-oligosaccharides, galacto-oligosaccharides, and trans-galacto-oligosaccharides. The fermentation of prebiotics by the GM results mainly in the production of SCFAs, which directly modulate acidity in the colon (79), intestinal epithelium development (80) and immune system regulation (81). Iyama et al. reported that including a combination of glutamine, fiber and oligosaccharides in the diet during allo-HSCT decreased the severity of the intestinal mucositis and was associated with higher overall survival at day + 100 compared to the control. Furthermore, this diet was associated with a significant reduction in translocation of *Enterococcus* species from the GM to the bloodstream (82). Yoshifuji et al. administered resistant starch and a prebiotics mixture containing glutamine, polydextrose, and lactosucrose from prior to conditioning to day 28 after allo-HSCT. They observed shortened duration of oral mucositis and diarrhea and reduced cumulative incidence of aGvHD grades 2–4, with a particular decrease of skin aGvHD in the prebiotic group compared to an historical cohort. GM diversity, relative abundance of butyrate-producing taxa and fecal butyrate concentration were preserved by prebiotics intake (83).

Another interesting dietary compound that could be administered is Lactoferrin. Lactoferrin is an iron-binding protein of bovine and human milk involved in iron homeostasis, with pleiotropic functions such as antimicrobial and immunoregulatory activity. Preclinical models showed that Lactoferrin seems to reduce bacterial translocation, improving GM eubiosis (84). In preterm neonates, bovine Lactoferrin administration reduced the incidence of late-onset sepsis and necrotizing enterocolitis improving eubiosis, strengthening the intestinal epithelial barrier and promoting antagonism toward enteropathogens (85–87). However, clinical evidence in other settings remain scarce. Rota et al. reported that symptoms of gut GvHD disappeared soon after lactoferrin therapy was started in an HSCT patient (88) highlighting the potential role of lactoferrin administration in transplant recipients. Interestingly, in a randomized controlled trial of pediatric patients receiving induction chemotherapy for hematologic malignancies, oral lactoferrin supplementation promoted the maintenance of diversity during chemotherapy and was associated with reduced outgrowth of pathobionts, like *Enterococcus* (89).

### Probiotics

Probiotics are defined as “live microorganisms which, when administered in adequate amounts, confer a health benefit on the host” according to the Food and Agriculture Organization of the United Nations and World Health Organization (90), and could be also found in traditional and commonly eaten foods. Probiotic use among cancer patients undergoing treatment is increasing (91), with *Lactobacillus* spp. and *Bifidobacterium* spp. being the most utilized (92). The administration of living organisms in patients with hematologic malignancies and altered gut permeability is still controversial due to safety concerns. Various authors reported cases of BSI or sepsis caused by probiotic bacterial strains (93–95). However, Ladas et al. reported that administration of *Lactobacillus plantarum* in a cohort of 30 pediatric patients is safe and feasible, with no bacteremia or adverse events reported (96). A Phase I clinical trial to evaluate the safety and tolerability of Clostridium butyricum CBM 588 Probiotic Strain administration during HSCT (from day + 1 to + 28) is ongoing (NCT03922035). Moreover, the use of fungal probiotics such as *Saccharomyces boulardii* CNCM I-745 has been explored, but its use remains controversial, since several cases of fungemia were reported (97, 98).

A diet richer in fiber and probiotics prior to transplant was associated with earlier neutrophil engraftment and a shorter duration of febrile neutropenia in a small observational study (99). In a randomized, non-placebo-controlled trial 20 HSCT recipients were supplemented with *Lactobacillus rhamnosus GG*. No appreciable probiotic-related change in the GM or difference in the incidence of GvHD were observed (100). To date, given the uncertain safety profile and the lack of clinical evidence, the benefit of probiotic supplementation during allo-HSCT still needs to be determined. Moreover, in patient receiving immune checkpoint inhibitors for melanoma, a diet richer in fiber without probiotic administration was associated with improved outcome compared to probiotic use (101), suggesting that also in the HSCT setting other strategies rather than single probiotics could be prioritized for nutritional modulation of the GM.

### Synbiotics and post-biotics

Synbiotic are defined as a combination of probiotic bacteria and growth-promoting prebiotic that achieve “synergism” (102). The clinical application of synbiotic in the onco-hematological settings is limited. In an animal model of leukemic mice with cachexia, Bindels et al. reported that a synbiotic containing inulin-type fructan and *L. reuteri* 100–23 promoted the restoration of *Lactobacillus* species and the reduction of *Enterobacteriaceae*, with a positive effect on intestinal homeostasis and a prolonged survival (103).

Postbiotics are a heterogeneous group of functional bioactive compounds, defined as a preparation of inanimate microorganisms and/or their components that confers a health benefit on the host. They therefore contain inactivated microbial cells or cell components, with or without metabolites (104). Since they do not contain any living organism, they could be an attractive option for immunocompromised patients, where safety of live-bacterial products is still matter of concerns. In mouse models, the administration of exopolysaccharide derived from *Bacillus subtilis* ameliorated aGvHD improving GvHD related mortality. This effect was mediated by the induction of inhibitory dentritic cells in a TLR4-dependent manner (105).

SCFAs are the most investigated, and they represent a effector route in common with prebiotic compounds. Given the strong evidence associating increased GM-production of SCFA with protection from chronic GvHD (106), and acute GvHD (107), their administration could be explored in the near future, even if the challenge of delivering sufficient amounts of SCFA to the human colon is still present (108).

## Conclusion

We summarized the present evidence regarding the role of nutrition in the modulation of the GM in allo-HSCT recipients. Detailed investigations on the biological mechanism of nutritional intervention have been provided for the role of lactose, but still lacks for other dietary compound and for the use of EN vs. PN.

We believe that the following three areas of research should receive the most attention in the near future: the exact impact of which enteral formula is used for providing EN still needs to be evaluated, and lessons should be learned from dietary modulation applied in the management of IBD (109).

Several other prebiotics and postbiotics are currently under investigation in preclinical or clinical models, such as Lactoferrin, Inulin and Exopolysaccharide, and could be supplemented together with oral or enteral feeding. To date, several clinical trials evaluating the safety and efficacy of specific dietary elements for GM modulation in allo-HSCT recipients are ongoing, as summarized in Table 2. Other compounds that should receive attention are bile acids. Prophylactic administration of ursodeoxycholic acid is associated with reduced incidence of hepatic complications and severe aGvHD, and lower non-relapse mortality (110), and this clinical benefit could be partially explained by its effect on the GM (21, 25, 111). Therefore, studies analyzing the impact of bile acids administration on the metabolomic and metagenomic configuration of the GM are awaited.

**TABLE 2:** Ongoing trial for GM modulation in HSCT.

Nutrient	Setting	Diseases	NCT	Status
Resistant starch, Maltodextrin	Auto-HSCT adults	Myeloma or lymphoma	NCT05135351	Recruiting
Pre-biotic foods/drinks	Allo-HSCT adults	Any	NCT04629430	Recruiting
Clostridium butyricum CBM 588 probiotic strain	Allo-HSCT adults	Any	NCT03922035	Active, not recruiting
2’-fucosyllactose	Allo-HSCT pediatric	Any	NCT04263597	Recruiting
Inulin	Allo-HSCT pediatric	Any	NCT04111471	Recruiting
Human lysozyme goat milk	Allo-HSCT adults	Any	NCT04177004	Recruiting

Moreover, food consumption after the neutropenic phase should be strictly monitored, in order to unravel the potential relationship between consumed foods and GM modifications. Therefore, since evidences of the influence of single nutrients on HSCT outcomes are gathering, it would be interesting to analyze the impact of specific foods as well. All these interventions could provide effective, safe and cost effective ways to modulate the GM in order to improve clinical outcomes. A change in nutritional support during HSCT is awaited in the next years considering the growing evidence regarding its positive microbiological and clinical impact.

## Author contributions

EM designed the work. DL, FB, and FV wrote the manuscript. AP and RM critically revised the manuscript. All authors contributed to the article and approved the submitted version.
